# Understanding the Artificial Intelligence Revolution and its Ethical Implications

**DOI:** 10.1007/s11673-025-10427-6

**Published:** 2025-08-14

**Authors:** Amin Beheshti, Ian Kerridge

**Affiliations:** 1 Macquarie University, Sydney, NSW Australia; 2 The University of Sydney, Sydney, NSW Australia

**Keywords:** Intelligence, Artificial intelligence (AI), Generative AI, Ethics of AI

## Abstract

Recent artificial intelligence (AI) advancements have precipitated profound ethical deliberations and societal concerns. These developments redefine the parameters of technology’s role in our daily lives and challenge our understanding of ethics in the context of AI-enabled processes. As AI systems become more integrated into various facets of human activity, from healthcare to finance and from social interactions to governance, the ethical implications of these technologies have become increasingly complex and pressing. In this paper, we aim to facilitate the understanding of intelligence and Artificial Intelligence and delve into the transformative impact of the AI revolution on societal norms and ethical frameworks. We spotlight the critical ethical questions and concerns that arise as AI technologies become increasingly embedded in various aspects of human life. We provide a brief overview of ethical strategies in AI development and explore how implementing these strategies can mitigate potential risks, promote responsible innovation, and ensure the alignment of AI technologies with societal values.

## I. Introduction

In recent years, the landscape of technology and its intersection with society has been profoundly reshaped by advancements in Artificial Intelligence (AI). These developments have expanded the horizons of technological possibilities, resulting in a new era of ethical deliberation and concern (Shaw 2019). As AI technologies become a part of our daily lives, it is crucial to carefully and thoroughly explore their ethical issues. In this context, it is important to understand what AI is and recognize how AI fundamentally changes how we think about ethics in society.

As AI systems grow more sophisticated and capable, their role in shaping human experiences, decisions, and interactions becomes more critical and influential. This evolution challenges traditional ethical paradigms, necessitating re-evaluating how we understand responsibility, autonomy, and the moral obligations of both creators and users of AI technologies (Etzioni and Etzioni 2017). To thoroughly explore these pressing issues, this paper is structured to facilitate understanding intelligence, beginning with analysing human intelligence’s analytical, Applied, and generative dimensions (Beheshti 2023). This exploration is a foundation for understanding the parallels and divergences between human intelligence and its artificial counterparts. By presenting the characteristics and capabilities of Analytical AI, Applied AI, and Generative AI, we illuminate how these technologies mirror, complement, and extend human intellectual capacities.

Furthermore, this examination extends to the ethical landscape in which these AI systems live. Accordingly, we delve into the ethical implications of integrating AI into various aspects of human life. We examine the challenges in ensuring these technologies are developed and deployed in ways that align with societal values and norms. By highlighting the critical ethical questions and concerns that emerge from the increasing presence of AI, we aim to contribute to the ongoing dialogue on responsible innovation in AI development.

This paper outlines the ethical dilemmas AI poses and highlights the ethical strategies in AI development. It emphasizes the importance of implementing measures that mitigate potential risks, foster responsible innovation, and ensure the alignment of AI technologies with society’s core values and ethical principles. Through a comprehensive overview of these strategies, we advocate for a path forward that balances technological advancement with ethical integrity, paving the way for AI to contribute positively to society without compromising moral standards.

In particular, this paper endeavours to enhance the understanding of intelligence and Artificial Intelligence, shedding light on the ethical complexities introduced by the AI revolution. By bridging the gap between technological innovation and ethical considerations, we aspire to contribute to the development of AI systems that not only push the boundaries of what is possible but do so in an ethically sound and socially responsible manner. The rest of the paper is organized as follows: Section II explores the concept of intelligence. In Section III, we delve into the specifics of Artificial Intelligence. Section IV examines the ethical implications arising from advancements in AI technologies. We then discuss ethical strategies for AI development in Section V before concluding the paper with remarks for future directions in Section VI.

## II. Understanding Intelligence

Intelligence is the capability to acquire and apply Knowledge and Skills to accomplish a Process (Beheshti 2023, 2022). A process is a structured set of interrelated tasks and activities designed to achieve specific goals, solve problems, and respond to challenges, encompassing the ability to adapt dynamically based on new information or changing circumstances. To deepen the understanding of “process” in the context of intelligence, it is essential to recognize that processes are not limited to static or predefined sequences of actions. Instead, they are dynamic and adaptable, capable of evolving in response to new information, changing environments, and unforeseen challenges. For example, adaptation to change—one of the hallmarks of intelligence—can itself be considered a process. This involves continuously assessing the situation, identifying deviations from expectations, and executing adjustments to maintain or improve performance. Similarly, creative problem-solving can be viewed as a process, encompassing the iterative exploration of ideas, synthesis of knowledge, and evaluation of potential solutions. By framing these complex capabilities as processes, we emphasize that intelligence operates through structured, goal-directed activities that are inherently flexible and responsive to diverse circumstances. This perspective highlights the versatility of the process framework in capturing the multifaceted nature of intelligent behaviour.

There are three main types of intelligence: (i) Analytical Intelligence, which is associated with the knowledge dimension, representing the capacity for analysis, evaluation, and comprehension of complex information; (ii) Applied Intelligence, which correlates with the skills dimension, indicating adeptness in acquiring and applying learned competencies; and (iii) Generative Intelligence, which corresponds to the capability dimension, reflecting the aptitude for creating innovative solutions and concepts. Intelligence is pivotal to executing processes, providing the necessary capability to acquire knowledge and skills for achieving targeted outcomes. In this context, a process comprises a series of tasks and activities designed to accomplish a specific objective (Beheshti et al. 2016). Examples include a personal process (e.g., goal setting, nutrition planning, or exercise routines) or a business (e.g., Patient Admission and Discharge Process or Insurance Claims Processing).

A. Analytical Intelligence
  Analytical intelligence, as it pertains to the component of “knowledge” within the broader definition of intelligence—the capability to acquire and apply knowledge and skills to accomplish a process—can be defined as the facet of analytical function that underscores an individual’s ability to process, analyse, and synthesize information. Analytical intelligence significantly depends on the accumulation of knowledge an intelligent entity acquires over time through observation and education. This aspect of intelligence is crucial for effectively absorbing and integrating new information, enabling individuals to discern relevant data, comprehend complex concepts, and seamlessly incorporate new insights into their existing knowledge frameworks. The essence of analytical intelligence lies in its application: utilizing logical reasoning, critical thinking, and adept problem-solving skills to dissect situations, identify viable solutions, and execute informed decisions. It encapsulates the capacity for breaking down complex issues into simpler, manageable components, recognizing patterns or connections, and strategizing systematically towards resolution. Furthermore, analytical intelligence involves critical evaluation of alternatives, balancing the pros and cons of different actions, and opting for the most logical, evidence-based choice.
B. Applied Intelligence
  Applied intelligence, as it pertains to the component of “skill” within the broader definition of intelligence—the capability to acquire and apply knowledge and skills to accomplish a process—can be specifically understood as the subset of intelligence that focuses on decision-making and choosing the best next steps. Applied intelligence encapsulates the capacity to hold information and, more critically, analyse, synthesize, and apply it effectively in various contexts. Thus, applied intelligence plays a pivotal role in enabling individuals to learn from experience, adapt to new situations, understand abstract concepts, and employ reasoning in pursuing goals, thereby driving the successful application of knowledge and skills in achieving desired outcomes.
  Applied AI is intricately connected to the cognitive aspects of intelligence, as it embodies the processes of acquiring, analysing, and applying knowledge to make informed decisions. Cognitive functions such as reasoning, problem-solving, and adaptive learning are central to Applied AI, enabling systems to process abstract concepts, synthesize diverse data, and dynamically respond to new situations. By leveraging cognitive principles, Applied AI mirrors the human ability to evaluate complex scenarios, draw from past experiences, and select optimal next steps to achieve specific goals. This alignment underscores how Applied AI operationalizes applied intelligence, transforming theoretical understanding into practical, goal-oriented outcomes across various contexts.
C. Generative Intelligence
  Generative intelligence can be conceptualized as the capability of a system to produce novel, valuable outputs by leveraging acquired knowledge and skills in ways that were not explicitly programmed or anticipated by its creators. This form of intelligence is characterized by the system’s ability to generate new content, ideas, solutions, or outputs that adapt to new situations, challenges, or requirements, effectively demonstrating creativity and innovation. Generative intelligence in humans is intrinsically linked to a suite of cognitive capabilities that drive ideation, innovation, and adaptation. At its core, ideation is the birthplace of creativity, allowing the mind to formulate concepts and possibilities. Reasoning, however, provides a logical structure to these ideas, enabling individuals to navigate complex problems and make sound decisions. Problem-solving is an active process where generative intelligence is applied to overcome obstacles, often requiring a blend of critical thinking and adaptability.

## III. Understanding Artificial Intelligence

Artificial Intelligence (AI) is defined as the development and implementation of computer systems or machines that can acquire and apply Knowledge and Skills to accomplish a Process (Beheshti 2023; Beheshti et al. 2023). Considering this definition, we have three main types of AI systems: Analytical AI, Applied AI, and Generative AI.

A. Analytical AI
  This AI system primarily focuses on understanding data and knowledge, mirroring the human ability to process, analyse, and synthesize information for effective problem-solving and decision-making (Beheshti et al. 2016a, b). Analytical AI stands at the forefront of technological advancement, embodying the essence of AI’s capability to acquire and apply knowledge and skills to accomplish complex processes.
  The core of Analytical AI lies in its sophisticated algorithms and computational processes designed to understand the data and transform it into contextualized data and knowledge (Russell and Norvig 2010). These AI systems excel in parsing large datasets, discerning patterns, identifying relationships, and extracting actionable insights from the information overload that characterizes the digital age. By doing so, Analytical AI enables the conversion of raw data into understandable and useful knowledge, facilitating informed decision-making across various domains, including healthcare, education, finance, environmental science, and beyond.
  Moreover, analytical AI’s strength is its ability to critically evaluate and systematically break down complex issues into simpler components for analysis. This involves recognizing patterns and critically assessing alternatives, weighing the pros and cons of potential solutions, and making decisions based on logical, evidence-based analyses (Russell and Norvig 2010). Such capabilities make Analytical AI instrumental in enhancing the efficiency of decision-making processes, optimizing operations, and driving innovation by providing previously unattainable insights.
  As we delve deeper into the age of Big Data (Rajabi and Beheshti 2016), the advancements in Analytical AI reflect a significant portion of AI research and development efforts. These advancements underscore the technology’s capacity to understand and analyse data at an unprecedented scale and apply this knowledge in creating solutions that address real-world challenges. Analytical AI extends the boundaries of what machines can accomplish and provides a valuable tool for humans to leverage to make more informed, rational, and impactful decisions in an increasingly complex world.
  For example, analytical AI in healthcare manifests as advanced diagnostic algorithms that interpret medical images with high precision, predictive analytics for real-time patient monitoring to pre-empt critical conditions, and personalized medicine by analysing genetic data for customized treatments. It also improves healthcare operations by optimizing hospital workflows and managing resources efficiently. These applications demonstrate the transformative impact of Analytical AI, enhancing patient care, treatment outcomes, and the efficiency of healthcare services by converting vast amounts of medical data into actionable insights and informed decisions.
B. Applied AI
  Within the vast and evolving landscape of artificial intelligence, “Applied AI” represents a ground-breaking approach that draws its core principles from the human faculty of Applied intelligence. Defined by its capacity to acquire, process, and apply knowledge, Applied AI epitomizes the advanced subset of AI systems dedicated to enhancing decision-making and orchestrating strategic next steps. This type of AI is intricately designed to emulate the myriad cognitive skills inherent in humans, such as perception, memory, reasoning, problem-solving, and decision-making, echoing the “skills"component integral to the broader definition of intelligence.
  Applied AI systems, including cognitive assistants and co-pilots, are engineered to go beyond mere data analysis; they aim to understand, learn from, and interact with their environment to mirror human cognitive processes (Zhao et al. 2022). These systems are adept at navigating the complexity of vast datasets, extracting meaningful patterns, and leveraging this information to facilitate informed decision-making. By doing so, they serve as invaluable tools in various contexts, from personalized recommendations and customer service to strategic planning and operational optimizations in businesses.
  The core advantage of Applied AI lies in its ability not just to process information but to synthesize and apply it effectively across different scenarios. This enables such systems to offer tailored advice, predict outcomes, and automate decision-making processes, thus acting as sophisticated advisors to their human counterparts. Integrating Applied AI into decision-making frameworks significantly enhances the capacity for analytical thinking, strategic planning, and problem-solving, making complex decision processes more efficient and effective.
  For example, in the healthcare domain, Applied AI systems are revolutionizing how knowledge workers, such as physicians, nurses, and clinical researchers, approach decision-making and patient care. In daily clinical practice, Applied AI systems act as advanced decision-support tools that can process the latest medical research, guidelines, and protocols. By keeping healthcare professionals abreast of the newest evidence-based practices, these AI systems ensure patient care decisions are informed by the most current and comprehensive medical knowledge available.
C. Generative AI
  Generative AI emerges as a transformative category inspired by the human capacity for generative intelligence. By innovatively applying acquired knowledge and skills, it is defined by its remarkable ability to produce novel and valuable outputs encompassing text, images, audio, programmes, and more. Unlike conventional AI systems bound by the limits of their programming, Generative AI transcends these boundaries, showcasing an unparalleled level of autonomy, flexibility, and creativity. t highlights the core strengths of generative intelligence and demonstrates its wide-ranging usefulness, from content creation and design to drug discovery and other complex tasks (Feuerriegel et al., 2024).
  Generative AI systems stand at the cutting edge of technology, driving the frontier of what artificial intelligence can achieve. They leverage complex algorithms and models, such as Generative Adversarial Networks (GANs) (Cresswell et al. 2018), to assimilate and recombine information in unprecedented ways, producing outcomes not explicitly predefined by their creators. This capability not only underscores the systems’ utility across various applications—from content creation and design to drug discovery and beyond—but also reflects human intelligence’s dynamic, adaptive nature.
  Generative AI systems are not merely tools but collaborators that enhance human creativity, offering new perspectives and possibilities that extend beyond the conventional scope of programmed algorithms (Chui et al. 2023). Generative AI represents a significant leap forward, bridging the gap between machine execution and human creativity. Its development and refinement continue to push the boundaries of AI, challenging our understanding of creativity and the potential for machines to replicate and contribute to the creative process. Thus, generative AI stands as a cornerstone in the ongoing advancement of AI technologies, heralding a future where the collaborative synergy between human and artificial intelligence fosters unprecedented levels of innovation and creative expression.
  For example, in the healthcare domain, Generative AI is revolutionizing the landscape with its ability to automate and enhance various processes. For instance, it is instrumental in drug discovery. AI systems like Generative Adversarial Networks (GANs) can predict molecular responses and generate new compounds, accelerating the pace at which new medications are developed and brought to market. Generative AI contributes to advancing diagnostic precision in medical imaging by creating highly detailed and accurate representations of medical scans. These AI-generated images can be used for training purposes or to augment sparse datasets, improving machine learning models without compromising patient privacy.
  Another exemplary application is in personalized medicine. Generative AI algorithms can assimilate vast amounts of genetic information to tailor treatments to an individual’s unique genetic makeup, optimizing the effectiveness of therapeutic interventions. Additionally, in synthetic data generation, Generative AI plays a pivotal role by creating anonymized healthcare data, which preserves patient confidentiality while providing researchers with rich datasets for epidemiological studies and clinical trials.
  In surgical planning and simulation, Generative AI systems can generate 3D models of patient anatomy, allowing surgeons to plan and practice complex procedures in a risk-free virtual environment. This improves the surgeon’s skill and reduces the likelihood of complications during actual surgeries. Furthermore, AI-enabled chatbots and virtual health assistants, powered by generative capabilities, can provide patients with personalized health advice and support, enhancing patient engagement and adherence to treatment protocols.

## IV. Ethical Implications of Advancements in AI Systems

The rapid evolution of AI technologies, spanning from Analytical AI to Applied AI and Generative AI, has not only captivated the imagination of the technological world but also sparked widespread debate and concern across all levels of society. Integrating these AI systems into daily processes raises profound ethical questions that demand new frameworks of thought and evaluation. As we delve into the ethical implications of these diverse AI systems, several pivotal considerations emerge, necessitating a comprehensive examination of the moral landscape shaped by AI (Zhao et al. 2022; Chui et al. 2023; Ho ¨glund and Khedri 2023).

A. Ethical Considerations in Analytical AI
  Analytical AI, focusing on understanding data and knowledge, prompts us to question the ethical boundaries of data utilization and the potential for bias in decision-making processes. While sophisticated, Analytical AI systems rely on the patterns and correlations present in their training data, which may reflect existing societal biases. This raises critical concerns about fairness, inclusivity, and the perpetuation of discrimination through flawed analytical models. Ensuring transparency and explainability in these systems is paramount, as it enables stakeholders to understand and challenge AI-driven decisions when necessary. Moreover, accountability mechanisms must be established to delineate responsibility for outcomes produced by Analytical AI.
B. Ethical Considerations in Applied AI
  Applied AI, designed to facilitate decision-making and emulate human cognitive processes, introduces another layer of ethical complexity. These systems often operate in real-world contexts, such as healthcare, finance, and law, where decisions can have far-reaching consequences. Key ethical challenges include ensuring the safety, reliability, and security of these systems, protecting privacy, and balancing AI autonomy with human oversight. Applied AI’s capacity to engage in what appears to be independent reasoning raises the question of whether these systems should be equipped to perform ethical reasoning and, if so, how to ensure alignment with human values. By incorporating ethical frameworks into the design and deployment of Applied AI, we can mitigate risks and enhance societal trust in these technologies.
C. Ethical Considerations in Generative AI
  Generative AI, known for its ability to produce novel content, pushes the boundaries of creativity while presenting unique ethical challenges. Questions of authorship, intellectual property, and the value of human versus AI-generated creations are central to the ethical discourse on Generative AI. Furthermore, the potential for these systems to generate harmful or misleading content, such as deepfakes or misinformation, underscores the need for accountability and responsible use. Safeguards, including content verification systems and legal frameworks, must be established to prevent misuse while fostering innovation in generative technologies.
D. An Integrated Ethical Framework for AI
  To address these diverse ethical challenges, a holistic ethical framework is essential—one that spans Analytical, Applied, and Generative AI. This framework should prioritize fairness, transparency, accountability, and respect for human rights across all types of AI. Drawing inspiration from interdisciplinary perspectives in philosophy, law, computer science, and social sciences (Mittelstadt 2019), the development of AI systems must align with societal values and ethical principles.
  Stakeholder involvement, including public awareness and input, is crucial in shaping the moral trajectory of AI technologies. This collective effort requires collaboration across disciplines, ensuring that AI enhances the human experience while safeguarding the moral fabric of society. By embedding ethical reasoning into the design, deployment, and regulation of AI, we can navigate the ethical complexities of AI’s integration into various domains and ensure its contributions are both innovative and ethically sound.

## V. Ethical Strategies in AI Development

To address the ethical considerations raised by the advancement of AI technologies, it is imperative to integrate features and frameworks that ensure responsible development, deployment, and use of AI systems. The following suggestions aim to prepare for and navigate the ethical dimensions associated with AI, particularly concerning its thinking structures, ethical reasoning capabilities, and implications across human activity (Mittelstadt 2019; Konda 2022; Stahl and Stahl 2021).

**Transparent and Explainable AI Models.** Developing AI systems with transparency and explainability at their core can help demystify the “structures of thinking” employed by these technologies. This involves creating models humans can understand and interpret, facilitating insight into how decisions are made and ensuring that AI systems can be audited for fairness, bias, and ethical considerations.

**Incorporation of Ethical Reasoning Capabilities.** AI systems can be designed to incorporate ethical principles and reasoning capabilities to address whether and how AI should engage in ethical reasoning. This could involve embedding ethical decision-making frameworks within AI algorithms, enabling them to consider ethical implications and outcomes in their processing and outputs. Ethical AI frameworks can be based on widely accepted ethical theories and principles tailored to the specific application areas of the AI system.

**Diverse and Inclusive Training Data.** Ensuring that AI systems are trained on diverse and inclusive datasets can help mitigate biases and enable more equitable outcomes. This involves actively seeking out and including underrepresented data in training to ensure AI systems have access to a broad spectrum of human cognition and experiences.

**Multi-stakeholder Engagement and Oversight.** Engaging a wide range of stakeholders in developing and deploying AI systems ensures that multiple perspectives are considered, especially those of communities most affected by AI technologies. This could include setting up ethics boards or committees representing various sectors, including ethicists, technologists, affected communities, and policymakers, to oversee AI projects and ensure they adhere to ethical standards.

**Regulatory and Ethical Standards Compliance.** Developing and adhering to rigorous regulatory and ethical standards is crucial for guiding AI’s ethical development and use. This includes establishing international and industry-specific guidelines and standards for ethical AI and mechanisms for enforcement and compliance checking.

**Continuous Ethical Training and Evaluation.** AI systems should undergo continuous ethical training and evaluation throughout their lifecycle. This involves regularly updating AI models with new data reflecting evolving societal norms and values and re-evaluating AI systems for ethical implications as they adapt and learn.

**Public Awareness and AI Literacy.** Promoting public awareness and understanding of AI technologies, including their potential ethical implications, is essential for fostering informed dialogue and decision-making. Educational initiatives and transparency about how AI works, its limitations and its impact on society can empower individuals to engage with AI technologies critically.

**Ethical AI Design Principles.** Adopt design principles that prioritize ethics from the inception of AI projects. This includes principles like harm prevention, fairness, privacy protection, and accountability. Embedding these principles into the design process encourages the creation of AI systems that inherently respect ethical standards.

**Stress Testing and Scenario Analysis.** Regularly conduct stress tests and scenario analyses to evaluate how AI systems might behave in extreme or unforeseen situations. This can help identify potential ethical issues or negative outcomes before they occur in real-world applications.

**Dynamic Consent Mechanisms.** Implement dynamic consent mechanisms for users, especially in applications that heavily interact with personal data or may affect personal decisions. This allows users to control what data is used and how it is used, ensuring respect for autonomy and consent.

**Human-in-the-Loop Systems.** Incorporate human oversight in AI decision-making processes, especially in critical areas like healthcare, legal systems, and financial services. Reinforcement Learning with Human Feedback (RLHF) and Reinforcement Learning from AI Feedback (RLAIF) techniques ensure that human experts vet AI recommendations before taking significant actions. In particular, implementing robust feedback mechanisms that allow users and affected parties to report ethical concerns or unintended consequences of AI systems can be invaluable for continuous improvement and adjusting AI systems to align with ethical expectations.

**Global Cooperation on AI Ethics.** Encourage international cooperation to establish global norms and standards for AI ethics. Given the borderless nature of AI technology and its impacts, international collaboration can help harmonize ethical standards and practices, ensuring a unified approach to addressing AI’s ethical challenges.

## VI. Conclusion

The intersection of Artificial Intelligence (AI) with various aspects of human life heralds a new era of innovation, efficiency, and opportunity. As AI systems become increasingly sophisticated and pervasive, addressing the ethical considerations they raise is paramount to ensuring these technologies serve the greater good. By embracing ethical strategies in AI development, we can navigate the complexities of modern AI applications while safeguarding human rights, dignity, and societal values. As we move forward, the collaborative efforts of technologists, ethicists, policymakers, and the broader community will be crucial in shaping an AI-enabled future that is not only technologically advanced but also ethically sound and socially responsible.

## Data Availability

Not applicable.
